# Inoculation Site from a Cutaneous Melanoma Patient Treated with an Allogeneic Therapeutic Vaccine: A Case Report

**DOI:** 10.3389/fimmu.2015.00144

**Published:** 2015-03-30

**Authors:** Mariana Aris, Alicia Inés Bravo, María Marcela Barrio, José Mordoh

**Affiliations:** ^1^Centro de Investigaciones Oncológicas-Fundación Cáncer (CIO-FUCA), Ciudad Autónoma de Buenos Aires, Argentina; ^2^Unidad de Inmunopatología, Hospital Interzonal General de Agudos Eva Perón, San Martín, Provincia de Buenos Aires, Argentina; ^3^Laboratorio de Cancerología, Fundación Instituto Leloir, IIBBA-CONICET, Ciudad Autónoma de Buenos Aires, Argentina; ^4^Instituto Médico Especializado Alexander Fleming, Ciudad Autónoma de Buenos Aires, Argentina

**Keywords:** cutaneous melanoma, allogeneic therapeutic cell vaccine, local injection reaction site, vaccination site biopsy immune profiling, local Ag presentation

## Abstract

We have developed a therapeutic vaccine consisting of a mixture of lethally-irradiated allogeneic cutaneous melanoma cell lines with BCG and GM-CSF as adjuvants. The CSF-470 vaccine is currently being assayed in a Phase II–III trial against medium-dose IFN-α2b. All vaccinated patients immunized intradermally developed large edematous erythema reactions, which then transformed into subcutaneous nodules active for several months. However, vaccine injection sites were not routinely biopsied. We describe the case of a female patient, previously classified as stage III, but who, due to the simultaneous discovery of bone metastases only received one vaccination was withdrawn from the study, and continued her treatment elsewhere. This patient developed a post-vaccination nodule which was surgically removed 7 weeks later, and allowed to analyze the reactivity and immune profiling of the inoculation site. An inflammatory reaction with zones of fibrosis, high irrigation, and brisk lymphoid infiltration, primarily composed of CD8^+^ and CD20^+^ lymphocytes, was observed. There were no remaining BCG bacilli, and scarce CD4^+^ and Foxp3^+^ T cells were determined. MART-1 Ag was found throughout the vaccination site. CD11c^+^ Ag presenting cells were either dispersed or forming dense nests. Some CD11c^+^ cells proliferated; most of them contained intracellular MART-1 Ag, and some interacted with CD8^+^ lymphocytes. These observations suggest a potent, long-lasting local inflammatory response with recruitment of Ag-presenting cells that incorporate melanoma Ags, probably leading to Ag presentation to naïve T cells.

## Introduction

A 45-year-old Caucasian woman (patient #1) underwent resection on May 2012 of a spontaneously bleeding congenital nevus in her back. Histological analysis revealed an ulcerated nodular cutaneous melanoma (CM) with a Breslow thickness of 7.8 mm, epithelioid and spindle cell morphology, non-brisk immune infiltration, and satellitosis. Concurrent adenopathies were detected in her right axilla; axillary lymph node dissection revealed 4/23 metastatic nodes. After signing informed consent on August 23, 2012, this high-risk patient underwent the routine scanning procedure of the CASVAC-0401 study; on the basis of a normal CAT scan on June 28, 2012, and normal laboratory, the patient was classified as stage III and randomized to the vaccine arm. The patient only received one dose of vaccine (16 × 10^6^ lethally irradiated allogeneic CM cells, plus 10^6^ cfu of Bacillus Calmette Guerin (BCG) and 400 μg of recombinant human granulocyte macrophage-colony stimulating factor (rhGM-CSF) in four daily doses), since already at the first visit (September 03, 2012) she complained of lumbar and left rib cage pain. Presence of bone metastases were suspected and were confirmed by a PET scan (October 03, 2012), which revealed bone metastases at the ninth left rib, the right acetabulum as well as soft tissue metastases at the right infra-axillary region. The patient was therefore already at stage IV of her disease, she was discontinued from the study and continued appropriate treatment elsewhere. The bone metastases were irradiated, and she started treatment with Vemurafenib since her tumor had the BRAF^V600E^ mutation. Seven weeks after the single vaccination, the patient decided to remove her subcutaneous (s.c.) vaccination nodule. The patient developed progressive disease, including brain metastases, and died on January 15, 2014.

**Figure 1:**
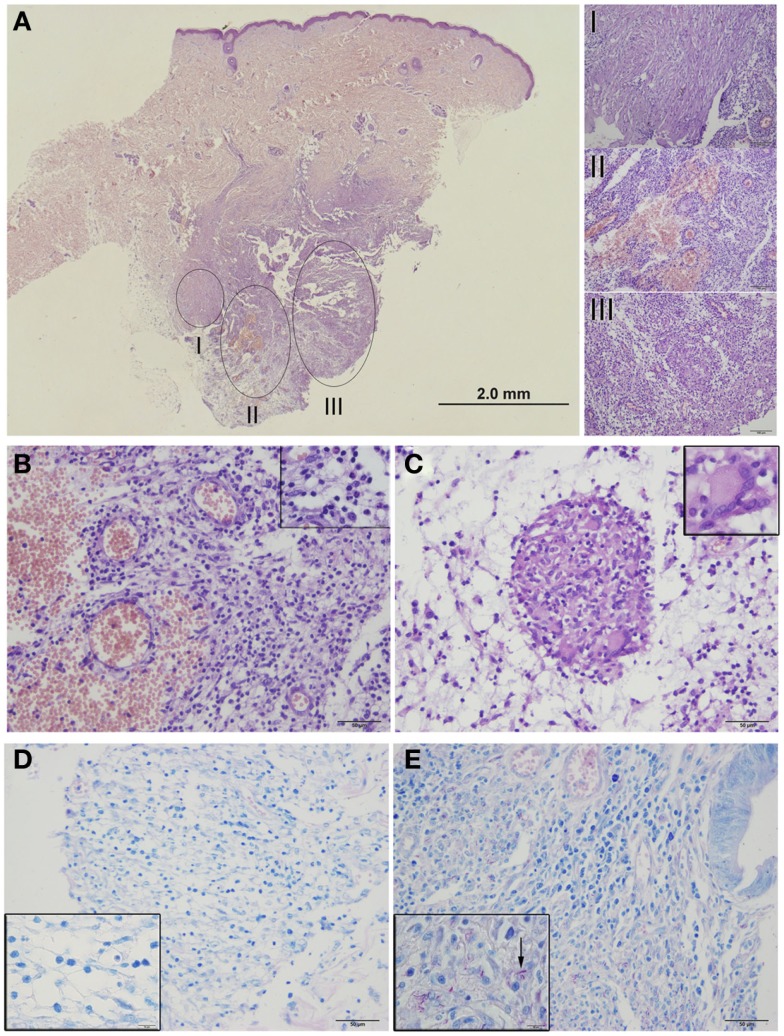
**Histological analysis of CSF-470 vaccination site biopsy from patient #1**. Hematoxylin–Eosin stained sections were examined by optical microscopy (Olympus BX40, Tokyo, Japan); pictures were acquired with Olympus Digital Camera DP72 and analyzed with Image J software. **(A)** Low magnification image of the granulomatous nodule distinguished a fibrosis zone (I), a highly vascularized zone (II), and a brisk-infiltrated with inflammatory cells zone (III). **(B)** Zone (II), in detail, showing lymphocytes and polymorphonuclear cells. **(C)** Zone (III) showing dense nested structures with a polynuclear cell (inset). **(D)** Ziehl–Neelsen staining revealed absence of BCG bacilli in the vaccine site. **(E)** A positive control for bacilli staining is shown (bowel tuberculosis). Bars = 2 mm **(A)**; 50 μm **(B–E)**.

**Figure 2:**
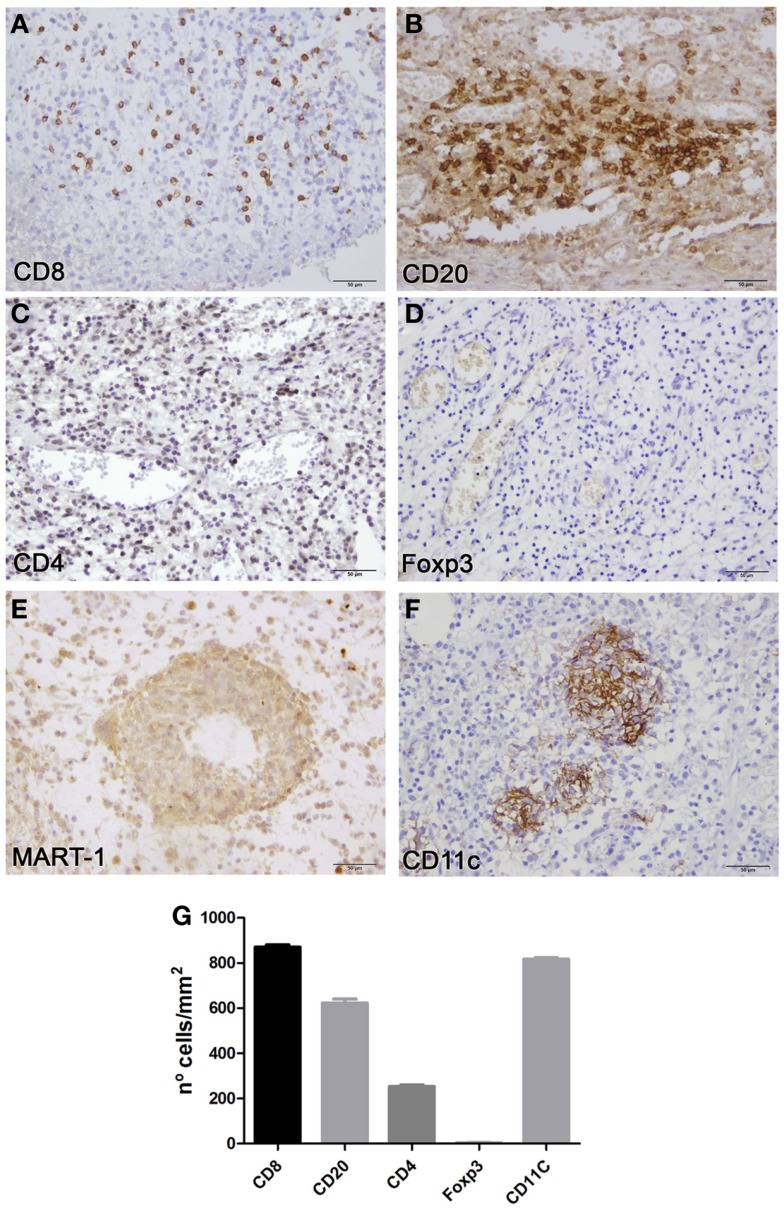
**Immune profiling and MART-1 distribution in CSF-470 vaccination site biopsy from patient #1**. Formalin-fixed, paraffin-embedded tissue sections were stained with appropriate antibodies, amplified with avidin-biotin-peroxidase (ABC) system (Vectastain, Vector Labs), and revealed with 3,3′-diaminobenzidine. Brisk CD8^+^ and CD20^+^ lymphocyte infiltration was observed in zones (II, III), with scarce CD4^+^ or Foxp3^+^ infiltration **(A–D)**. Expression of MART-1 Ag was observed throughout the inoculation site **(E)**. CD11c^+^ cells were observed in dense nested structures **(F)**. Quantification of the immune infiltrate (n° cells/mm^2^ tissue surface, mean ± SD, two determinations) **(G)**. Bars = 50 μm. Antibodies: CD8 (clone C8/144, Dako, CA, USA), CD20 (clone L26, Dako), CD4 (clone 1F6, Novocastra, Wetzlar, Germany), Foxp3 (clone 236A/E7, Abcam, MA, USA), MART-1 (clone A103, Dako), CD11c (clone EP1347Y, Abcam).

**Figure 3:**
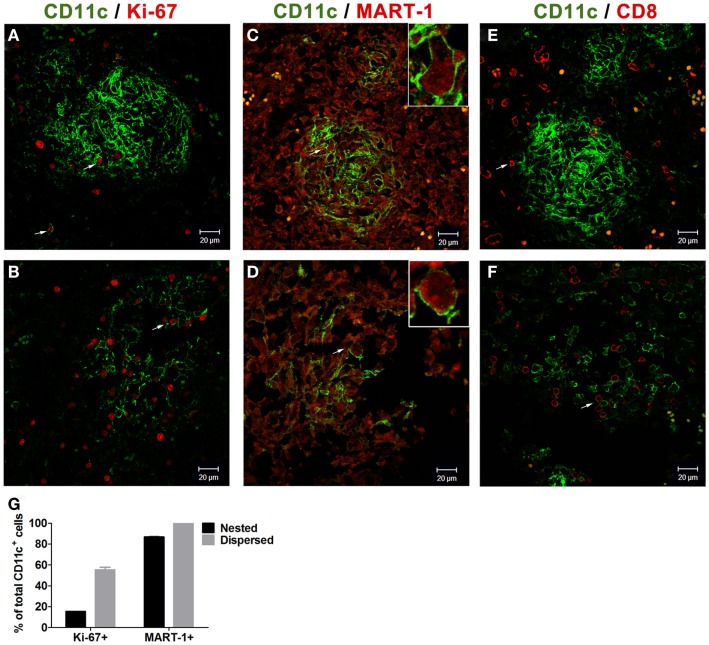
**Analysis of CD11c^+^ Ag presenting cells in CSF-470 vaccination site biopsy from patient #1**. Tissue sections were stained with appropriate antibodies, revealed with fluorophore-conjugated secondary antibodies, and examined by confocal microscopy (LSM 5 Zeiss Pascal, Oberkochen, Germany). Pictures were acquired with Zeiss LSM Image software and analyzed with Image J software. Both nested **(A)** and dispersed **(B)** CD11c^+^ cells showed some proliferating Ki-67^+^ cells. Most CD11c^+^ APCs incorporated CM Ag MART-1 [**(C–D)**, inset]. CD8^+^ lymphocytes were observed surrounding APCs nests, with some T cells interacting with nested **(E)** and dispersed CD11c^+^ cells **(F)**. Quantification of Ki-67^+^ and MART-1^+^ cells in nested and dispersed CD11c^+^ cells (mean ± SD, three high power fields, two determinations) **(G)**. Bars = 20 μm. Antibodies: Ki-67 (clone MIB-1, Dako); MART-1-AF647 [clone 2A9 (1)]. Fluorophore-conjugated secondary antibodies (Jackson Immunoresearch).

Routine analysis of vaccine nodules is not contemplated in the CASVAC-0401 study; this case offered the possibility to study the histology of one such nodule and its associated immune profile. After formaldehyde fixation and paraffin embedding of the entire sample, the vaccination site biopsy was thoroughly analyzed. The s.c. nodule was located 2 mm below the epidermis (Figure 1A) and three distinct areas of fibrosis, high vascularization, and brisk lymphoid infiltration could be distinguished (Figure 1AI–III). Lymphocytes and polymorphonuclear cells were distributed around vessels (Figure 1B). Dense nested structures comprised of macrophages, histiocytes and polynuclear cells, typically found in inflammatory processes including responses to BCG, were observed in the highly infiltrated zone (Figure 1C). BCG was rapidly cleared from the site, since no remaining bacilli were detected by Ziehl–Nielsen staining at the vaccination site (Figures 1D,E). Immune profiling analysis revealed brisk CD8^+^ and CD20^+^ lymphocyte infiltration, non-brisk CD4^+^, and scarce Foxp3^+^ T cells infiltration (Figures 2A–D,G). MART-1 antigen (Ag), derived from the vaccine, was found throughout the inflammatory focus (Figure 2E). Brisk infiltration of CD11c^+^ Ag-presenting cells (APCs) was also observed; these cells were either dispersed in the dermis or assembled in multiple dense nested structures (Figures 2F,G). Further analysis revealed that some CD11c^+^ APCs, both dispersed and nested, proliferated (Figures 3A,B,G), most had phagocytosed MART-1 Ag (Figures 3C,D,G), and some were surrounded by CD8^+^ lymphocytes (Figures 3E,F), suggesting local Ag presentation. The near absence of FoxP3^+^ lymphocytes also suggests that an immunogenic environment was created.

## Background

Cutaneous melanoma is a prototypic immunogenic tumor for which several immunotherapeutic approaches are currently under investigation (2). Therapeutic cancer vaccines are aimed at promoting tumor-specific and long-term immunity. Cancer vaccines have been assayed using different strategies such as the use of inactivated whole tumor cells, Ag-specific peptides or purified proteins, among others, in combination with adjuvants to create an immunogenic microenvironment for Ag presentation and expansion of cytotoxic T lymphocytes (3). The classic paradigm proposes that following inoculation in the dermis, Ags are incorporated and processed by APCs, such as macrophages and dendritic cells (DCs), which then migrate to lymph nodes (LN), where processed Ags are presented to naïve T lymphocytes (1). However, the vaccination system of choice, combining suitable Ags, adequate adjuvants, and an appropriate immunization schedule is a delicate equation that may give rise to tolerance or immunogenicity. Therefore, dissection of the events that take place at the vaccination site is important to unravel the induction of an effective immune response.

We have developed multi-Ag allogeneic vaccines for CM treatment. The CSF-470 vaccine, consisting of four lethally irradiated allogeneic CM cell lines plus BCG and rhGM-CSF as adjuvants, is currently being assayed versus medium-dose IFN-α2b (2:1 ratio) in a randomized open trial (CASVAC-0401) in stages IIB, IIC, and III post-surgery CM patients in adjuvancy (Clinical trials.gov NCT01729663). The study has been approved by the *Comité de Etica en Investigación del Instituto Médico Especializado Alexander Fleming*, and it has so far recruited 32 patients (21 patients in the vaccine arm and 11 patients in the IFN-α2b arm). The rationale for the use of this formulation is to immunize patients with an inert scaffold that spans a broad repertoire of tumor Ag, thus counteracting heterogeneity in Ag expression (4). Adjuvant BCG induces a potent local inflammatory reaction with a T_H_1-polarizing immune response and epitope spreading (5); rhGM-CSF stimulates local attraction of APCs and favors a T_H_1 response (6). In a previous Phase I study with escalating GM-CSF dosage, 400 μg rhGM-CSF per vaccine was found to be the optimal dose. The combination was safe and induced a predominantly cellular immune response (7).

## Discussion

The results presented here constitute the first *in situ* evidence of the afferent arm of the immune response to our cell-based melanoma vaccine. In every patient of the vaccine arm, a three phase local reaction took place: (i) large erythema (typically 10 cm diameter) with a smaller edema (typically 3 cm diameter) at the injection site which started on day 2 and lasted about 5–7 days; (ii) this local reaction later transformed into an erythematous papule, which occasionally ulcerated, and lasted several weeks; (iii) the dermal reaction subsided but a subcutaneous nodule persisted, lasting several months. Other trials with autologous non-small-cell lung tumor vaccines and GM-CSF reported development of erythemas with indurations following immunization, supporting these findings (8, 9). The histological and immune profiling of the vaccination site biopsy of patient #1 revealed a potent local inflammatory response, including highly irrigated and infiltrated zones. The CSF-470 vaccine is comprised of allogeneic lethally irradiated CM cells; thus, a *host versus graft* rejection reaction is expected at the vaccination site, fueled by BCG and GM-CSF. Interestingly, we could detect *in situ* affluence of CD11c^+^ APCs that incorporated MART-1 Ag and interacted locally with CD8^+^ cells, persisting for several weeks after vaccination (Figure 3). This suggests that Ag presentation takes place at the immunization site. Although the classic paradigm proposes that Ag presentation occurs *exclusively* in secondary LNs, evidence from afferent lymph vessels in normal human skin revealed an increased number of mainly memory/effector CD4^+^CD45Ro^+^ T cells and IL-12^+^ DCs in contact with IFN-α-producing T cells, supporting that T cells may be stimulated by APCs in peripheral tissues (10). Additionally, tertiary lymphoid structures (TLS) can arise in non-lymphoid organs during chronic inflammation, as seen in autoimmune responses, graft rejection, atherosclerosis, microbial infection, and cancer (11). TLS can generate effector and memory T cells that lead to allograft rejection (12). We have described the development of TLS in mice following repeated immunization with DC loaded with apoptotic/necrotic B16 melanoma cells, supporting the important role of local events in the generation of a systemic anti-tumor immune response (13).

We know from murine models that graft infiltration by host inflammatory monocytes and DCs is a hallmark of graft rejection; after which APCs travel to the LNs and cross-prime CD8^+^ T cells, the main effectors of graft rejection (14). Previous reports on allogeneic GM-CSF-secreting tumor cell vaccines in patients with pancreatic adenocarcinoma showed affluence of mononuclear and eosinophilic cells at the vaccination site on day 3 and increased CD3^+^ cells infiltration on day 7 (15). In other study with prostate cancer patients, immunization led to recruitment of CD1a^+^ DCs, CD68^+^ macrophages, eosinophils, and neutrophils 4 days following inoculation; CD4^+^ and CD8^+^ cell affluence increased with immunizations (16). Pioneering work from Dranoff *et al.* showed that immunization with lethally irradiated autologous tumor cells transfected with GM-CSF induced specific immune responses in several pathologies including CM, leukemia, and brain cancer (6, 17, 18). Immunization with this formulation in CM patients revealed intense infiltration with T lymphocytes, DCs, macrophages, and eosinophils (6). Examination of the vaccination site here described indicates that a potent immune reaction is taking place, which lasts several weeks, and that it has all the cellular ingredients that might lead to a positive immune response against tumor Ags.

## Concluding Remarks

The vaccine inoculation site is the gateway for the induction of an immune response. In this study, we gained access to a vaccine site biopsy from a CM patient following a single immunization. We propose that the generation of an immune response toward tumor Ags by using allogeneic vaccines involves a potent local inflammation, driven by allograft rejection, and fueled by BCG and rhGM-CSF. This would favor a proper immunogenic environment for APC affluence, which subsequently may interact locally with Ags or migrate toward peripheral LNs for Ag presentation. Since the CASVAC-0401 study comprises a total of 13 vaccinations over a period of 2 years, it will be important to analyze the evolution of vaccination sites at several time points in an *ad hoc* Phase I trial.

## Conflict of Interest Statement

The authors declare that the research was conducted in the absence of any commercial or financial relationships that could be construed as a potential conflict of interest.

## Abbreviations

Ag/Ags, antigen/antigens; APC, Ag presenting cells; BCG, bacillus calmette guerin; CM, cutaneous melanoma; DC, dendritic cells; LN, lymph node; rhGM-CSF, recombinant human granulocyte macrophage-colony stimulating factor; s.c., subcutaneous; TLS, tertiary lymphoid structures.
